# Atmospheric composition of exoplanets based on the thermal escape of gases and implications for habitability

**DOI:** 10.1098/rspa.2020.0148

**Published:** 2020-09-09

**Authors:** Samuel Konatham, Javier Martin-Torres, Maria-Paz Zorzano

**Affiliations:** 1 Group of Atmospheric Science, Department of Computer Science, Electrical and Space Engineering, Luleå University of Technology, Luleå, Sweden; 2 Instituto Andaluz de Ciencias de la Tierra (CSIC-UGR), Armilla, Granada, Spain; 3 School of Geosciences, University of Aberdeen, Meston Building, King's College, Aberdeen, UK; 4 Centro de Astrobiología (CSIC-INTA), Torrejón de Ardoz, Madrid, Spain

**Keywords:** habitability, exoplanets, atmospheres, kinetic theory, thermal escape

## Abstract

The detection of habitable exoplanets is an exciting scientific and technical challenge. Owing to the current and most likely long-lasting impossibility of performing *in situ* exploration of exoplanets, their study and hypotheses regarding their capability to host life will be based on the restricted low-resolution spatial and spectral information of their atmospheres. On the other hand, with the advent of the upcoming exoplanet survey missions and technological improvements, there is a need for preliminary discrimination that can prioritize potential candidates within the fast-growing list of exoplanets. Here we estimate, for the first time and using the kinetic theory of gases, a list of the possible atmospheric species that can be retained in the atmospheres of the known exoplanets. We conclude that, based on our current knowledge of the detected exoplanets, 45 of them are good candidates for habitability studies. These exoplanets could have Earth-like atmospheres and should be able to maintain stable liquid water. Our results suggest that the current definition of a habitable zone around a star should be revisited and that the capacity of the planet to host an Earth-like atmosphere to support the stability of liquid water should be added.

## 1. Introduction

Since the discovery of PSR B1257 + 12b [1] in 1992, the number of known exoplanets has increased rapidly. With the present and future generations of space-based and ground-based instruments, this number is expected to increase significantly soon. Besides the fundamental interest of compiling an extensive catalogue of exoplanets, finding a subset of habitable worlds would be of utmost importance from a strategic point of view. In fact, owing to the current and most likely ever-lasting impossibility of performing *in situ* exploration of exoplanets, studies on their habitability will be based on the restricted, remote observations of their atmospheres and spectral detections of potential biosignatures [2]. Developing a way to prioritize observed exoplanets based on their likelihood of being habitable through atmospheric modelling and orbits in habitable zones around stars will save effort and time for the scientific community.

The formation of the atmosphere of a planet starts with the accretion of available gases from the protoplanetary disc, and the atmospheric species currently observed depend on its evolution afterwards. Molecules transported through chondritic and nebular elements and dissolved on the early magma through accretion processes contribute to the initial formation of the planet, forming part of its core [3]. The planetesimal and gas accretion rates during the accretion process and the subsequent contraction of the gas envelope under the influence of gravity contribute to the internal energy budget of the planet [4]. Furthermore, during the initial conditions of planet formation, the metallicity of the protoplanetary disc determines the abundance of available volatiles [5]. High metallicity and migration of protoplanets were found to facilitate a high incidence of giant planets compared with the metal-poor stars [4]. Overall, planet formation and the availability of heavy elements in the primordial atmosphere are closely related to the metallicity of the protoplanetary disc.

Geological processes such as volcanic eruptions lead to a secondary atmosphere [6]. The ulterior evolution of a planet's atmosphere is determined by other available sources and sinks, the atmospheric chemistry, the presence and strength of a magnetic field [7] and the incident radiation from the host star. Over the geological periods, the atmospheric escape mechanisms play a significant role in shaping the atmospheric composition and its evolution. The escape of atmospheric species affects the atmospheric chemistry, which in turn impacts the habitability of exoplanets.

In 1846, Waterston [8], for the first time, presented a model dealing with the thermal escape of light gases from the Earth's atmosphere. Stoney [9] later improved this model by explaining the escape of H_2_ and He from the atmosphere of the Earth and the absence of atmosphere on the Moon. Significant improvements have been made to the thermal escape problem ever since [10–13], including other forms of atmospheric escape mechanisms such as Jeans escape, thermodynamic escape and several non-thermal escape mechanisms that are possible in a planetary atmosphere.

Atmospheric escape in exoplanet atmospheres has attracted much attention from the scientific community after the detection of the hydrodynamic escape of atmospheric species from the atmosphere of hot Jupiter HD209458 b [14]. Since then, a multitude of sophisticated one- and multi-dimensional atmospheric models have been presented for exoplanets; these are comprehensively reviewed in [7,15,16]. These atmospheric escape models are predominantly designed for hot Jupiters and close-in, highly irradiated exoplanets that undergo mass loss through hydrodynamic blow-off. The Earth-like low-mass terrestrial exoplanets are more prone to experience slow thermal escape driven by thermal velocities of atmospheric species.

The thermal escape in atmospheres can be divided into slow thermal escape (Jeans escape or classical thermal escape) and hydrodynamic escape. The type of thermal escape is determined by the hydrostatic or non-hydrostatic nature of the exosphere [15]. Only a few atmospheric escape models are adapted to low-mass exoplanets such as super-Earths and sub-Neptunes, with a hydrodynamic upper atmosphere [15,17]. In summary, all exoplanet atmospheric escape models to date have been developed for hydrodynamic conditions and the hydrostatic upper atmospheres of terrestrial low-irradiated exoplanets is mostly unexplored except for the Jeans escape model.

The rate of escape of atmospheric species is influenced mainly by the activity and emitted radiation of the host star, which determines the atmospheric escape regime to be either a slow thermal escape or a hydrodynamic blow-off of the upper atmosphere [11,18]. The presence of the planetary magnetic field and its consequent interaction with stellar winds influences and determines the possible atmospheric escape processes. While the magnetic field does not significantly influence the thermal escape process constituting the escape of neutral gas species, other processes such as ion pick-up, sputtering and hydrodynamic escape rates are influenced by the strength of the planetary magnetic field [19,20]. In the case of hot Jupiters, numerical models indicate that the presence of a planetary magnetic field reduces the mass-loss rate [21]. Through early ingress during UV transits, the planetary magnetic field has been detected for hot Jupiter WASP-12 b [22].

The well-established Jeans escape model requires physical parameters describing the exosphere, column density and exobase temperature for calculations. The atmospheric retrievals using transit spectroscopy and photometry could constrain the brightness temperature and pressure–temperature profiles of exoplanet atmospheres. The degeneracies due to cloud/haze and reference pressure [23,24] along with lower sensitivity to terrestrial Earth-mass exoplanets inhibit knowledge of the exospheres. At present, the unavailability of exospheric and flux density information makes it impossible to consider the Jeans escape or the diffusion-limited escape mechanism to estimate the atmospheric composition of exoplanets. Our knowledge of these atmospheric parameters in the case of exoplanets is similar to or even less than the knowledge of these parameters for the planets of the Solar System at the end of the ninteenth century and the beginning of the twentieth century when the first thermal escape models were developed. Nevertheless, the results of those initial models were able to provide reasonable estimates of the atmospheric species of the Solar System planets, in particular the lack of atmosphere on the Moon and the loss of H and He from the atmosphere of the Earth [9].

Here we present a new atmospheric thermal escape model for exoplanets with hydrostatic upper atmospheres. From the fundamentals of the kinetic theory of gases, we estimate the possible atmospheric constituents. The results of our model will further provide a list of terrestrial low-mass exoplanet targets for habitability studies compiled from evaluating their possible atmospheric compositions driven by thermal escape. We incorporated a conservative approach in our calculations and, based on the known physical parameters of the exoplanets, we determine the upper limit on the mass of the species that can escape from their atmospheres, and then we estimate their possible atmospheric constituents.

## 2. Model

Thermal escape is a function of the temperature of the exosphere and escape velocity of the planet. The vertical temperature profile of the planet, the temperature of the host star and the gravity of the planet are the three most important parameters driving thermal escape in planetary atmospheres [4–12]. The gravitational potential of a planet restricts the gaseous species from escaping its atmosphere owing to their kinetic velocities (temperature of gas species). As a general guideline, the combination of high temperature and low gravity leads to more significant loss of atmospheric species than the combination of low temperature and high gravity. The latter results in thick atmospheres, as seen in the gas giants of the Solar System.

The Solar System planets have been well observed, probed and studied by the scientific community since the advent of the space age with multiple satellite and *in situ* missions and well-resolved remote sensing observations using ground-based telescopes. The observations have resulted in, broadly speaking, good knowledge of the atmospheric profiles and atmospheric compositions of the Solar System planets [25,26].

By contrast, for exoplanets, information about their atmospheres and compositions is hard to achieve (excluding a few close-in hot Jupiters) because of technological limitations, relatively limited observations and the astronomical distances involved. The atmospheric escape process and the resulting effluent flux of atmospheric gases is a result of a combination of multiple escape processes determined by the physical and chemical properties of the planet's atmosphere. The non-thermal escape mechanisms such as photochemical escape, ion pick-up, sputtering, the polar wind, charge exchange and impact erosion should be examined on a case-by-case basis because of their dependence on atmospheric chemistry and abundances. By means of the understanding gained from the observations of non-thermal escape processes on Mars, Titan and other icy moons, one can study the non-thermal escape processes for exoplanets with prior knowledge of their atmospheric compositions and chemical interactions. Studies on non-thermal escape processes in exoplanet atmospheres are minimal owing to the dominance of hydrodynamic escape [15]. For a detailed and accurate study of exoplanet atmospheres, all possible atmospheric escape processes including thermal and non-thermal escape processes should be analysed.

The main objective of this paper is to develop a method for preliminary classification of the discovered exoplanets based on their ability to hold an atmosphere. This method can be applied using the values of mass, radius and equilibrium temperature that are available or deducible for most of the discovered exoplanets. The results from our model complement the existing exoplanet classifications by adding the atmospheric component by means of thermal escape analysis, i.e. the possible atmospheric compositions, especially for low-mass exoplanets. At this juncture, when the catalogue of exoplanets is increasing drastically with new space missions coming into effect, such a simple universal method can prioritize a shortlist of exoplanets for further habitability studies and observations. Therefore, we present the model using the fundamentals of the kinetic theory of gases, estimating the atmospheric compositions by determining the atmospheric species that can escape from the atmospheres of the exoplanets through thermal escape. Our model will further place constraints on the possible dominant species in the exoplanet atmospheres, limiting the atmospheric species that could shape the atmosphere.

The escape velocity of the planet is $V_{\text{esc}}=\sqrt{2GM\text{/}R}$, where *G* is a universal gravitational constant (6.6743 × 10^−11^ m^3 ^kg^−1^ s^−2^) and *M* (kg) and *R* (m) are the mass and radius of the planet, respectively. The values of *M* and *R* are available for the planets of our Solar System and a good number of confirmed exoplanets [27–29]. The thermal velocity (root mean square velocity) of the atmospheric species determines the rate of thermal escape of the species from the atmosphere. This is defined as $U=\sqrt{3k_{b}T\text{/}m}$, where *m* (kg) is the mass of gas, *T* (K) is the temperature and *k*_*b*_ (1.380 649 × 10^−23^ m^2 ^kg s^−2 ^K^−1^) is Boltzmann's constant.

The Jeans escape estimate of the escape flux, $\emptyset_{J}$, at the critical level (exobase) is

2.1 $$\emptyset_{J}=n_{c}B\frac{U}{2\sqrt{\pi }}(1+\lambda_{c})e^{-\lambda_{c}},$$

where the Jeans parameter, $\lambda_{c}$, is

2.2 $$\lambda_{c}={\left(\frac{V_{\text{esc}}}{U}\right)}^{2}=\frac{GMm}{k_{b}T_{c}R_{c}}=\frac{R_{c}}{H},$$

*n_c_* is the number density of the gas

2.3 $$n_{c}=n_{0}{\text{e}}^{\left(\lambda_{c}-\lambda_{0}\right)},$$

B={0.5−0.8} compensates for the repopulation of the high-energy tail of the Maxwell–Boltzmann distribution, *T_c_* (K) is the exobase temperature, *H* is the scale height, *R* is the radius of the planet and the subscript *c* represents the parameters at the exobase. $\lambda_{0}$ is the Jeans parameter at reference level (usually at the surface).

In the formalism of Jeans escape in planetary atmospheres, as a guideline, a factor of one-sixth is used conventionally to compare $U\;\text{and}\;V_{\text{esc}}$ in the atmospheric escape calculations. The origin of this factor is not known and does not represent any physical process in the underlying atmosphere, but it is generally accepted on an empirical basis that the escape process can happen if the thermal velocity (*U*) of the gas at the exobase is more than one-sixth of the *V*_esc_ [30,31].

The exobase of the exoplanets is not known, and we have no knowledge of the parameters $T_{c},\;n_{c}\;\text{and}\;\lambda_{c}$ in equation (2.1) of the exoplanet atmospheres. The exobase temperature varies with the parent star cycles and, in general, is significantly higher than the equilibrium temperature of the planet. On Earth, for example, the temperature of the exobase is typically around 1000 K, and the equilibrium temperature is about 255 K [26,30]. The altitudes and pressures of the exosphere are different at different locations on the planet. The temperature of the exosphere is influenced mainly by the radiative cooling agent present in the atmosphere. Generally, a CO_2_-dominant atmosphere will have cooler exospheric temperatures and H–He-dominant atmospheres have hotter exospheres [15]. Therefore, a linear or nonlinear relation between equilibrium temperature and exobase temperature can only be established using sophisticated radiative transfer models along with the knowledge of atmospheric compositions.

Because of the unavailability of the required parameters at the exobase and because the thermal escape of atmospheric species is driven by their thermal velocities, the equilibrium temperature can be the final resort to estimate slow thermal escape. In this work, we use the calculated equilibrium temperature of the exoplanets as a conservative approach to estimate and analyse the escape of the atmospheric species. The equilibrium temperature, *T*_*p*_ (K), of an exoplanet can be computed using the Stefan–Boltzmann law,

2.4 $${T_{p}}^{4}=\frac{\left(1-a\right)}{4}{\left(\frac{r}{d}\right)}^{2}{T_{\text{eff}}}^{4},$$

where *T*_eff_ (K) is the effective temperature of the star, *a* (unitless) is the bond albedo of the exoplanet, *r* (m) is the radius of the star and *d* (m) is the semi-major axis of the exoplanet. Owing to the unavailability of the albedo of the exoplanets, the equilibrium temperature *T*_*p*_ (K) is calculated by assuming zero albedo (*a* = 0).

**Figure 1.**
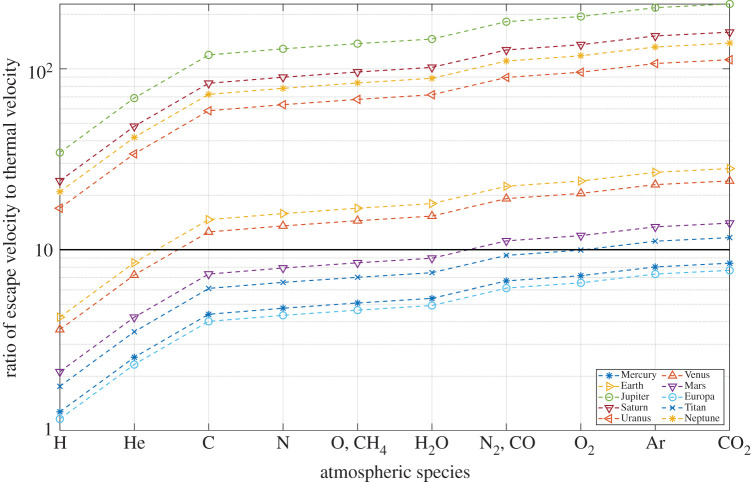
Ratio of escape velocity to thermal velocity of atmospheric species for Solar System objects. The solid horizontal line represents ratio = 10. (Online version in colour.)

As we use $T_{p}$, which is, in general, significantly lower than $T_{c}$, a factor lower than one-sixth must be chosen, which can appropriately represent the atmospheric escape process occurring from the higher layers of the atmosphere (exosphere). We have determined a suitable escape parameter, as shown in figure 1, by examining the ratio of the escape velocity of the Solar System objects to the thermal velocities of major atmospheric species at temperature $T_{p}$ calculated from equation (2.4).

In this paper, we consider atmospheric species with mass less than 44 g mol^−1^, including CO_2_ as the ‘major gases’. The gases are chosen based on their significance to the atmosphere and importance in facilitating biosignature gases. These include lighter atmospheric species from H and He and constitute most of the greenhouse gases CH_4_, CO_2_ and H_2_O along with six biogenic elements (CNOPSH) that are the building blocks of potential biosignature gases [32]. The biogenic compounds comprise the elements carbon (C), nitrogen (N), oxygen (O), phosphorus (P), sulfur (S) and hydrogen (H).

**Figure 2.**
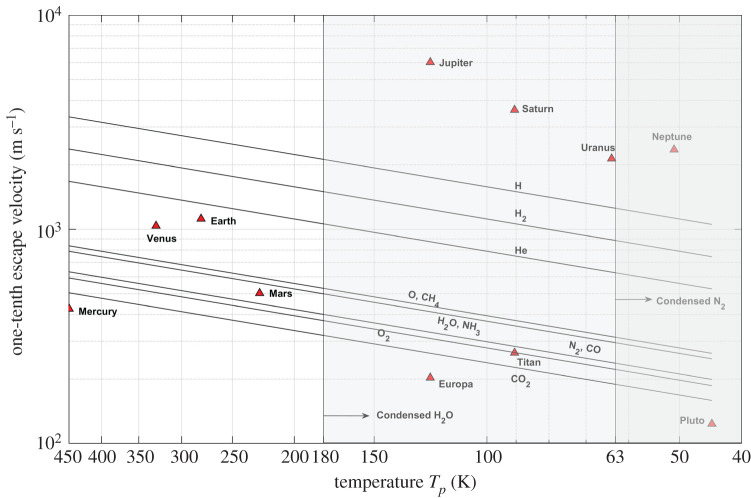
Atmospheric escape results for Solar System objects. The shaded regions to the left at 63 K and 180 K indicate the freezing temperatures of N_2_ and H_2_O, respectively, below which the species exist in the solid state. (Online version in colour.)

Table 1 shows the observed atmospheric species escaping from the atmospheres of Solar System objects by thermal and non-thermal escape mechanisms. Comparing the ratio of the velocities and the observed escaping gas species, we find that the ratio of velocities for each planet should be in the following range to match the observed atmospheres: Earth (8.5–17), Venus (7.2–14.5), Mars (9–11) and Titan (9.3–11.7). Collectively, these Solar System objects constrain the escape parameter within the range (9.3–11). Any value within this narrow range provides reasonable estimates for the Solar System objects. For simplicity, we have chosen a representative escape parameter of 10, which is the value used in previous thermal escape models [9]. The selected ratio of velocities is inferred from the detected escaping gases from the atmospheres of Solar System objects. Figures 1 and 2 and table 1, along with the subsequent text presenting the analysis of the results for Solar System objects, form the basis for selecting the one-tenth factor for this model. The results from the model are inherently permissive, considering the assumptions about albedo and the one-tenth factor. The results provide the upper bound of the atmospheric compositions and lower bound on the escaping atmospheric species. This ratio determines the upper bound on the mass of species that can escape from the atmosphere, including for those that are undetected.

**Table 1.** Known gases observed to escape from the Solar System planets and moons through *in situ* and remote sensing observations [30,33,34].

planet	escaping gases
Mercury	all major gases
Venus	H, He
Earth	H, He
Mars	H, C, O, N, Ar
Titan	H, CH_4_, N_2_
giant planets	no gases lost

The escape parameter represents the relative strength of the gravitational potential with respect to the thermal velocity. Varying the escape parameter within the constrained interval (9.3–11), greater and smaller than 10 corresponds to the weaker and stronger gravitational potential, respectively. For a given planet, the results obtained with an escape parameter greater than 10 will show more atmospheric escape, and results with a lower escape parameter will show weak atmospheric escape.

As observed from the results for the Solar System objects, our model is applicable and provides reasonable estimates for the terrestrial exoplanets undergoing slow thermal escape. Equation (2.5) shows the factored comparison of *U* and *V*_esc_ for the atmospheric species to escape the atmosphere of an exoplanet,

2.5 $$U>\frac{1}{10}V_{\text{esc}}.$$

Although only a small fraction of gas molecules that fall under the tail end of the Maxwell–Boltzmann distribution attain such high speeds, the gas can be removed slowly and consistently from the atmosphere over geological time scales.

Here we compute, for the known exoplanets, the thermal velocity of the gases and compare it with the escape velocity, as shown in equation (2.5), to determine if the gases can be retained in their atmospheres. The escape of the atmospheric species and dominant isotopes is dependent on their atomic/molecular weights and thermal velocities. The speed of axial rotation of the planet also affects the speeds of gas molecules at the higher altitudes of the atmosphere [9]. The speed of rotation of the planet influences the velocities of zonal winds, which in turn affect the global atmospheric circulation and temperature gradients in the atmosphere [35]. A fast-rotating planet produces high-velocity zonal winds that result in reducing the albedo of the planet, thereby increasing the equilibrium temperature [36]. The atmospheric species of a fast-rotating planet attain high velocities owing to increased temperature, thereby accelerating the atmospheric escape process.

For tidally locked exoplanets, the equilibrium temperature formulation does not give a complete picture as only the dayside of the exoplanet is perpetually exposed to the radiation from the host star and the heat transport mechanisms between the dayside and nightside determine the thermal contrasts. Nonetheless, some tidally locked exoplanets with synchronous rotation around the host star, which are capable of having liquid water on their surface, have been speculated to be potentially habitable [37,38]. With substantial implications, terrestrial exoplanets are expected to avoid synchronous rotation by atmospheric thermal tides. Thermal tide contributions from a tiny atmospheric envelope can maintain the exoplanet in an asynchronous rotation scheme around the host star [39]. On this basis, we do not include tidal locking of exoplanets in our model for the time being.

## 3. Results

### (a) Solar System planets and moons

Figure 2 shows the atmospheric escape of Solar System objects from the results of our model. The sloping lines in figure 2 indicate the thermal velocity of different atmospheric species as a function of the kinetic temperatures (referred to as velocity lines hereafter). A planet/moon is capable of retaining a particular atmospheric species in its atmosphere if the velocity line of the atmospheric species lies below the position of the planet/moon in figure 2 and, conversely, the atmospheric species escapes the atmosphere of the planet/moon if its velocity line lies above its position. The results for atmospheric escape for Solar System planets obtained from our model closely match those presented in [40] and the observed atmospheres.

The escape of different atmospheric species from the atmospheres is a result of multiple mechanisms involving stellar and atmospheric conditions. Although the problem is complex, the atmospheric composition can be estimated to some extent from the atmospheric thermal escape. This can be seen by applying the model to the objects of the Solar System (figure 2). An extensive literature on the atmospheric compositions and structures of the Solar System planets is available; this literature discusses in detail the formation and evolution of atmospheres. Here, we present in brief the observations of Solar System planets presented in detail by [26,31,33] that are relevant to our model.

The significance and the rate of mass loss from a strictly thermal or non-thermal escape process from the atmospheres are subject to multiple factors, including but not limited to radiation energy, atmospheric chemistry, elemental abundances, magnetic field strength and interactions with stellar winds. In our Solar System, the atmosphere of Earth is driven by a dominant thermal escape; on Mars, non-thermal escape mechanisms are more dominant [13,15]. Therefore, a direct relation such as the ratio of thermal escape to non-thermal escape cannot be established given the diversity of possible exoplanet atmospheres.

*H, H_2_, He.* As seen in figure 2, and as expected, the giant planets Jupiter, Saturn, Uranus and Neptune can accrete and hold all or most of the available gases in their atmospheres, starting from hydrogen (H), owing to their strong gravitational potentials and low temperatures (in agreement with the already known information). The runaway gas accretion and subsequent evolution during planet formation lead to dense H–He-dominant atmospheres. Inferring strictly from the equilibrium temperatures (approximately 100 K), these giant planets could cause condensation of most of the atmospheric species. However, the internal heat sources from the accretion process leading to high pressures and temperatures constrain the condensations. The terrestrial planets Mercury, Venus, Earth and Mars along with most of the moons in the Solar System are observed to lose H and He [9,13] because of a combination of low gravitational potential and high thermal velocities of these lighter gas species and non-thermal escape processes.

*CH_4_, NH_3_, H_2_O, O*. Along with the giant planets, Earth and Venus are known to retain oxygen and methane in their atmospheres. The planets Mercury and Mars are incapable of retaining these gases in their atmospheres. The controversial detections of trace amounts of methane (CH_4_), reported to be observed in the Martian atmosphere with seasonal variation, are attributed to surface or subsurface sources [41,42]. Figure 2 shows something that may look contradictory: that CH_4_, which is the well-known dominant species in Titan's atmosphere, can escape the atmosphere. CH_4_ is known to escape Titan's atmosphere through various mechanisms, including hydrodynamic escape, ion pick-up, sputtering and dissociation [43,44].

Nevertheless, although CH_4_ escapes from Titan's atmosphere, there is a hypothesized significant source of CH_4_ due to condensation and continuous supply from subsurface clathrates [45] that maintains the observed large amounts of liquid and gaseous CH_4_ found in its atmosphere. Similarly, Europa has been observed to have CH_4_ abundances attributed to the clathrates and condensing temperature [46]. Pluto, because of its low temperatures, has CH_4_ ice on its surface [47]. Jeans escape on Pluto is CH_4_ dominant [48]. Venus and Earth retain atmospheric species heavier than He. Mars is known to lose water vapour, thereby making it a trace gas in the atmosphere. This is attributed to escape mechanisms facilitated by the absence of a magnetosphere and low gravity, such as photodissociation, ion pick-up, ion outflow and sputtering [41].

*N_2_*. While the early evolution of Titan's atmosphere is still debated to this day, the accretion of NH_3_ in its early atmosphere was considered the primary source of N_2_ through photolysis [49], and the supply of volatiles from comets is also considered to be a possible source [50]. Continuous supply from these primordial sources from early formation times resulted in the large amounts of N_2_ observed in its present atmosphere. N_2_ escape from Titan's atmosphere is an existing process as a result of various mechanisms and has been observed (as well as CH_4_ escape) by Cassini in Titan's corona [51]. Because of its low gravitational potential, the Galilean moon Europa is incapable of holding most atmospheric species, including N_2_, in its atmosphere, leading to a tenuous atmosphere. By contrast, previous work simulating aqueous chemistry in the endogenic water–rock interactions indicates the possibility of compounds rich in nitrogen, carbon and hydrogen [52]. Europa, through plumes ejected from under its surface, loses water and heavier compounds [53]. Pluto and the Neptunian moon Triton have an N_2_-dominant atmosphere [48] and a suite of condensed atmospheric species on the surface due to cold temperatures.

*CO_2_*. Carbon dioxide is a significant greenhouse gas for the Solar System planets along with H_2_O and CH_4_. The atmospheres of the planets Venus and Mars are CO_2_ dominant, and Earth also has significant concentrations of CO_2_. Gases that are lighter than CO_2_ escape from the atmospheres of Mercury, Europa and Pluto, as shown in figure 2. The atmospheric mass loss for Mercury is a result of low escape velocity, high temperature and interactions with the solar wind. Although Pluto and Europa have very low escape velocities compared with the rest of the Solar System objects, their low temperatures facilitate condensation of the atmospheric species on the surface [47].

One of the main limitations of the kinetic theory of gases used in our model is the phase changes of the gas species determined by temperature and partial pressure, but the model still provides (as seen in figure 2) an excellent approach to the composition of the moons and planets of the Solar System. While partial pressure cannot be determined or commented upon, caution should be observed while inferring information on atmospheres with temperatures low enough to condense or freeze certain gas species. The observations of liquid CH_4_ on Titan and condensed CH_4_, NH_3_ and H_2_O ice on Europa, Pluto and Triton [45–48] indicate that the gas species which condense in an atmosphere would be retained by the exoplanet on its surface/subsurface. The correlation of our model results with the observed atmospheres of the Solar System shows that our approach is a simple way to create a shortlist of exoplanets to analyse and that further observations would improve our knowledge.

### (b) Exoplanets

**Figure 3.**
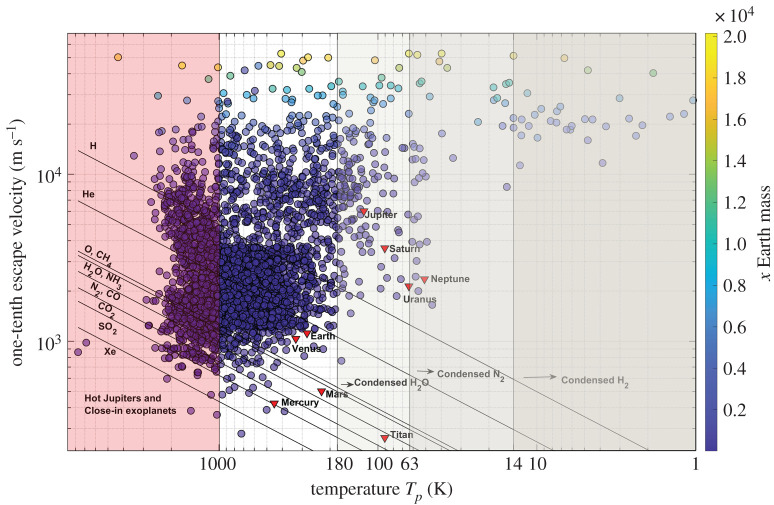
Diagram of one-tenth of the escape velocity versus the equilibrium temperature *T*_*p*_ of the exoplanets and Solar System planets/moons. The lines represent the thermal velocity of atmospheric species. The complete distribution in the graph shows a wide variety of exoplanets from cold giants on the left to hot Jupiters to the right, and small size exoplanets at the bottom and exoplanets of greater size at the top with the increasing height from the *x*-axis. The Solar System objects are also shown. The shaded regions to the left at 14 K, 63 K and 180 K indicate the freezing temperatures of H_2_, N_2_ and H_2_O, respectively, below which the species exist in the solid state. The region with temperatures above 1000 K indicates that the exoplanets are either hot Jupiters or close-in exoplanets. (Online version in colour.)

The constituents of the atmosphere and ability to retain them are of vital importance to the habitability of exoplanets [32,54]. An exoplanet capable of having an atmosphere and with the ability to hold biosignature gases is of more interest in habitability studies than exoplanets without atmospheres or evaporating/silicate atmospheres. Our model estimates the atmospheric species that can escape and those that can be retained in the atmosphere of the exoplanet. Figure 3 shows the thermal velocity of the atmospheric species as a function of the calculated equilibrium temperature of the exoplanets and their escape velocities. We have included the results for Solar System objects for comparison. The figure should be interpreted as follows: the exoplanets lying above the H velocity line have gravity strong enough to retain H and all atmospheric species heavier than H in their atmosphere. H escapes from the atmospheres of exoplanets placed between the H and He velocity lines as they can hold He and heavier atmospheric species in the atmosphere, leading to a possible He-dominant or other heavier gas-dominant atmosphere, and so on.

In figure 3, freezing temperatures below which the atmospheric species H_2_, N_2_ and H_2_O condense are highlighted in the shaded regions. The giant exoplanets orbiting very close to the host star with very short periods (hot Jupiters) have temperatures higher than approximately 1000 K [25]. In figure 3, the region with *T*_*p*_ > 1000 K is populated with very hot, giant exoplanets and close-in exoplanets like Mercury. The exoplanets with *T*_*p*_ < 180 K would have H_2_O in the solid or gaseous state depending on the surface temperature and atmospheric pressure. Similarly, the exoplanets with *T*_*p*_ < 14 K and *T*_*p*_ < 63 K would have H_2_ and N_2_ in either the solid or gas phase. The results are discussed in the sections below.

We have used our model to analyse 3705 exoplanets for which data required for the calculations are available (as of June 2019). We present our results using the mass-based exoplanet classification [27].

### (c) Comparison with atmospheric species data and limitations of the model

Our model, although it considers only the thermal escape of atmospheric species from atmospheres, serves the purpose of providing a preliminary classification of all exoplanets as the considered escape mechanism is prevalent for all types of exoplanets irrespective of their size.

**Figure 4.**
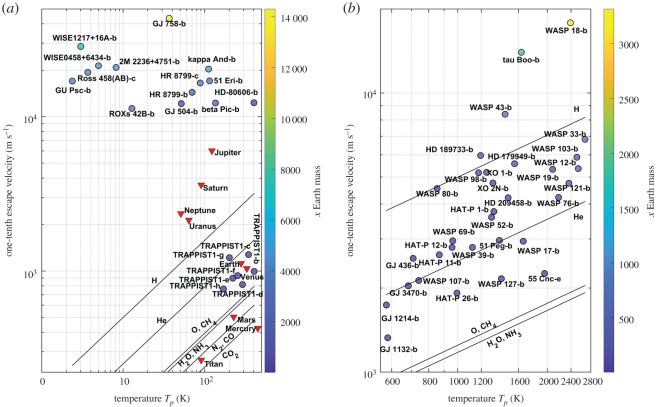
Estimates of the atmospheric escape from our model for the exoplanets on which atmospheric species were detected. One-tenth of the escape velocity–equilibrium temperature *T*_*p*_: (*a*) exoplanets with *T*_*p*_ < 500 K; (*b*) exoplanets with *T*_*p*_ > 500 K. (Online version in colour.)

Here we compare our results with the atmospheric species detections recorded to date in exoplanet atmospheres. Figure 4 shows the exoplanets for which detections of different atmospheric species were reported (references included in the electronic supplementary material). The figure shows the results in two panels divided as 0–500 K and greater than 500 K for ease of observing the results by including the names of the exoplanets. Comparing the results of the model with the reported gas detections in the exoplanet atmospheres, we find from figure 4*a* that the exoplanets with atmospheric detections and equilibrium temperatures less than 500 K are ice/gas giants and TRAPPIST-1 exoplanets. The model concludes that the cold gas giants can trap all gases, including hydrogen, and correlates with the reported H_2_, CH_4_, H_2_O, K, CO and N detections in their atmospheres. The TRAPPIST-1 system is of major significance as four of the seven exoplanets are orbiting in the habitable zone (HZ) of the star and are expected to have bulk compositions in a combination of rocky and water-enriched material [55]. The results from figure 4*a* indicate that the TRAPPIST-1 exoplanets are capable of retaining H_2_O in the atmosphere, concurring with the expected (undetected to date) presence of water on the TRAPPIST-1 exoplanets [55,56].

Figure 4*b* shows the exoplanets which are predominantly hot Jupiters and close-in exoplanets. The hot Jupiter exoplanets, similar to HD 209458 b, HD 189733 b, WASP 12 b and GJ 436 b, being formed close to their parent star, have high temperatures and experience hydrodynamic blow-off of the atmosphere [57–61]. For the exoplanets WASP 39 b and 51 Peg b from figure 4*b* we estimate that all atmospheric species heavier than He can be retained in the atmosphere and that these exoplanets are capable of retaining H_2_O in their atmosphere; detection of both He and H_2_O has been reported [62,63]. The exoplanet GJ 1132 b, owing to intense XUV radiation, is predicted to lose more massive atmospheric species along with H from the atmosphere [64].

For the exoplanets undergoing hydrodynamic escape, the estimates from our model do not concur with the detections of heavier atmospheric species in the extended atmospheres. The estimates of atmospheric species that can be retained in the atmosphere for exoplanets which do not experience hydrodynamic flow concur with the reported detections; this is demonstrated in figure 4 and electronic supplementary material, table S1, which shows the list of predicted and detected atmospheric species in 54 exoplanet atmospheres along with the estimates from our model of the gases that the exoplanets can hold in their atmosphere. The estimates of atmospheric species in exoplanet atmospheres from our model hold unless a hydrodynamic blow-off of the atmosphere is detected.

**Figure 5.**
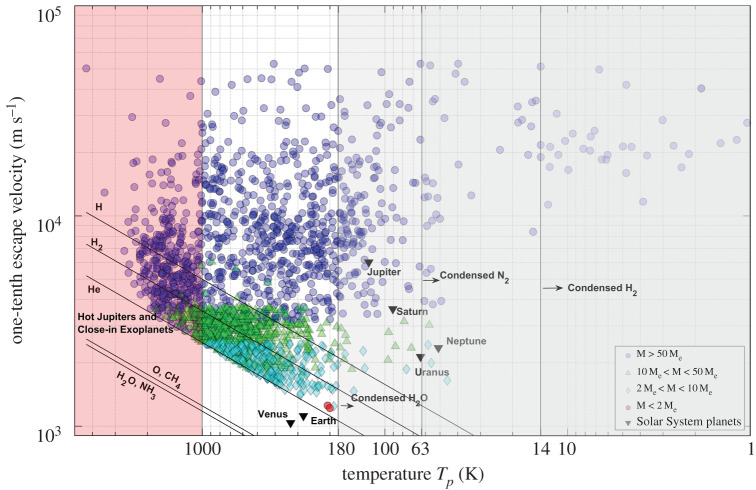
One-tenth of the escape velocity–equilibrium temperature, *T*_*p*_, diagram of exoplanets capable of H/He-abundant atmospheres and the giant planets of the Solar System. M is the mass of the exoplanet and M_e_ is the mass of Earth. The exoplanet classification we adopted shows the position of exoplanets in the graph with respect to their mass, indicating the type of exoplanets expected to have H or He in their atmospheres. The shaded regions to the left at 14 K, 63 K and 180 K indicate the freezing temperatures of H_2_, N_2_ and H_2_O, respectively, below which the species exist in the solid state. The region with temperatures above 1000 K indicates that the exoplanets are either hot Jupiters or close-in exoplanets. (Online version in colour.)

**Figure 6.**
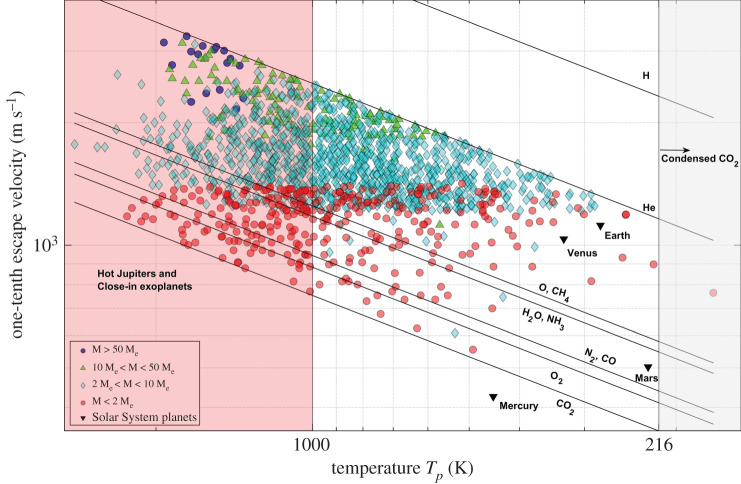
One-tenth of the escape velocity–equilibrium temperature, *T*_*p*_, diagram of exoplanets capable of an H_2_O/N_2_/CO_2_ atmosphere and the terrestrial planets of the Solar System. M is the mass of the exoplanet and M_e_ is the mass of Earth. This classification shows exoplanets of different masses and categories capable of holding carbon-, nitrogen- or water-rich atmospheres, as indicated in the correlation with terrestrial planets of the Solar System. Seventeen Jovian-sized exoplanets (shown in blue circles at the top right of the figure) are estimated to lose H and He from their atmospheres. The shaded regions to the left at 180 K and 216 K indicate the freezing temperatures of H_2_O and CO_2_, respectively, below which they exist in the solid state. The region with temperatures above 1000 K indicates that the exoplanets are either hot Jupiters or close-in exoplanets. (Online version in colour.)

We group the results for exoplanets into two classes based on their capability to retain atmospheric species lighter than CO_2_ in conjunction with exoplanet masses (figures 5 and 6). This approach is chosen in contrast to the general mass-based classification of exoplanets to differentiate the exoplanets with possible H–He-dominant and heavier gas (O_2_, CH_4_, H_2_O, NH_3_, N_2_ and CO_2_)-dominant atmospheres while also showing the distribution of Jupiter, Neptune or super-Earth-sized exoplanets across the diverse possibility of atmospheres.

### (d) Exoplanets capable of atmospheres with gases lighter than 44 g/mol (CO_2_)

Figure 5 shows the atmospheres of Jovian (M > 50 M_e_), Neptunian (10 M_e_ < M < 50 M_e_) and super-Earth (2 M_e_ < M < 10 M_e_) exoplanets along with two Earth-like (M < 2 M_e_) exoplanets. Because of their strong gravitational potential, they are capable of trapping in their atmosphere not only species with high mass but also the lightest atmospheric species such as H and He.

In figure 5, the exoplanets with equilibrium temperatures, *T*_*p*_ < 14 K and 14 K < *T*_*p*_ < 63 K, are too cold to have any gaseous species except for H and He. The formation of giant exoplanets following core accretion leading to runaway gas accretion [4] indicates that these giant exoplanets could accrete heavier gas species from the surrounding protoplanetary disc. The very low equilibrium temperatures of these giant planets suggest that these are either formed *in situ* or have experienced an outward migration [65].

The hot Jupiter exoplanets are considered to be migrated inward and supposedly experience hydrodynamic blow-off of the atmosphere, resulting in mass loss of heavier gases such as Na and K from the atmosphere in the process [66]. Hot Jupiters are outside the scope of this study owing to the strong hydrodynamic blow-off regimes of atmospheric escape, and high temperatures prevent them from being habitable.

H and H_2_ can escape from the exoplanets Kepler-186 f and TRAPPIST-1 g (red circles in figure 5) because they have masses close to 1.5 times that of Earth and are located in the habitable zones of their host stars [27]. Figure 5 shows a large population of Neptune- and Jupiter-sized exoplanets in the equilibrium temperature range 200–300 K. H_2_ could thermally escape from the atmospheres of some of the exoplanets in this population.

After H and He, the heavier and most significant atmospheric species are H_2_O, N_2_ and CO_2_. Figure 6 shows the exoplanets that are capable of holding atmospheric species heavier than He in the atmosphere, and that could lead to any of the H_2_O/N_2_/O_2_/CO_2_ being a dominant species in the atmosphere.

Any of the CO_2_, N_2_ or H_2_O species could be the principal constituent in the atmosphere of these exoplanets, depending on the atmospheric chemistry, processes and evolution. The exoplanets shown in figure 6 are predominantly super-Earth and Earth-like exoplanets that may have a rocky surface. The atmospheres of the exoplanets in this group are susceptible to the runaway greenhouse effect because of their capability to hold greenhouse gases like CO_2_, H_2_O and CH_4_ in the atmosphere. The exoplanets with an Earth-like atmosphere consisting of CO_2_ and H_2_O as greenhouse gases, which absorb more than 375 W/m^2^ from the stellar flux, will experience a runaway greenhouse effect, eventually evaporating all the surface water and ice with increasing surface temperatures [67].

Terrestrial planets of the Solar System, Venus, Earth and Mars fall under this category (as shown in figure 6), thereby making the exoplanets with similar temperatures and escape velocities strong candidates for potential habitability. From figure 6, we observe that the majority of super-Earth exoplanets placed between the He and O/CH_4_ velocity lines are capable of holding atmospheric species heavier than He in their atmosphere with few super-Earth exoplanets capable of losing H_2_O and O_2_ from the atmosphere because of lower escape velocities. The Neptunian exoplanets shown in figure 5 cannot hold He in their atmosphere because of high temperatures and low escape velocities compared with other Neptunian exoplanets seen in figure 5.

In figure 6, there are 17 Jovian exoplanets, which are listed in table 2. These exoplanets are hot Jupiters and are possibly undergoing hydrodynamic escape from the atmosphere.

**Table 2.** Jovian exoplanets that are capable of a possible H_2_O/N_2_/CO_2_ atmosphere.

exoplanet	mass (M_e_)	*T*_*p*_
HAT-P 47 b	65.5	1601
HAT-P 48 b	53.41	1358
HAT-P 65 b	167.55	1930
HAT-P 67 b	108.10	1508
HATS-19 b	135.76	1545
HD 76700 b	73.13	1438
K2 39 b	50.30	1861
KELT 11 b	62	1710
Kepler 7 b	137.67	1626
Kepler 12 b	137.03	1479
WASP 17 b	154.52	1659
WASP 20 b	99.52	1380
WASP 63 b	120.82	1531
WASP 127 b	57.23	1400
WASP 131 b	85.84	1459
WASP 153 b	124	1711
WASP 172 b	149.43	1745

### (e) Exoplanets capable of evaporating/tenuous atmospheres with gases heavier than 44 g/mol (CO_2_)

**Figure 7.**
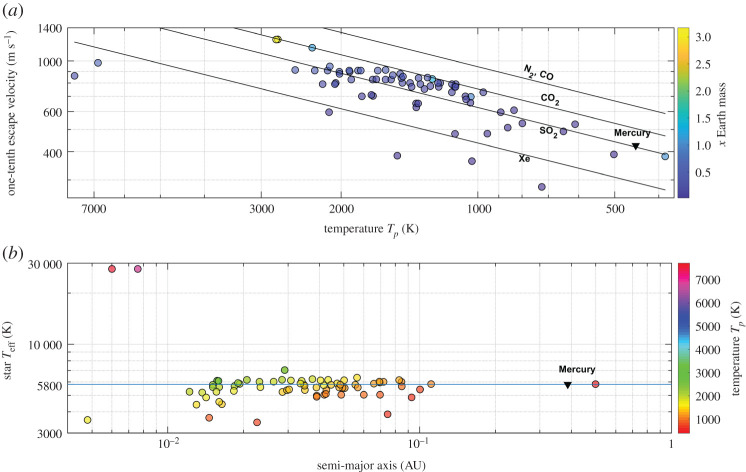
(*a*) One-tenth of the escape velocity–equilibrium temperature, *T*_*p*_, diagram of exoplanets capable of holding thin evaporating atmospheres. (*b*) The distance of exoplanets from the parent star versus the effective temperature of the star. The horizontal line at 5800 K in (*b*) indicates the Sun and the position of the planet Mercury is shown by a black triangle. The figure shows the low-mass exoplanets orbiting very close to the respective host star for which the resulting high temperatures combined with low gravitational potential result in loss of atmospheric species lighter than CO_2_. (Online version in colour.)

Most of the atmospheric species escape from the atmospheres of exoplanets with low gravitational potential and high temperatures, as observed in our Solar System with Mercury. Figure 6 shows the exoplanets that are incapable of retaining most of the atmospheric species, therefore developing an evaporating atmosphere [68]. Figure 7*a* shows the one-tenth escape velocity versus the equilibrium temperature along with the velocity lines of atmospheric species and figure 7*b* shows the distance of the exoplanet from the parent star and the temperature of the parent star. The exoplanets in figure 7 are close-in exoplanets that formed too close to their parent stars, rendering them inhospitable because of extreme temperatures and evaporating atmospheres.

### (f) Potentially habitable exoplanets

The HZ is defined as the region around a star where an exoplanet can have liquid water on its surface. The boundaries of the HZ around a star are estimated by considering a cloud-free, one-dimensional climate model and imposing moist-greenhouse and greenhouse limits [69]. The habitability of a planet is driven by the availability of an energy source, liquid solvent and nutrients for metabolic activity [2,54]. In this paper, we follow the definition of a habitable planet presented in [2] with liquid water as a solvent.

In this paper, we introduce the following criteria to consider an exoplanet as potentially habitable: (i) ability to host an Earth-like atmosphere and (ii) have equilibrium temperatures (assuming zero albedos) between 260 K and 320 K, i.e. able to host liquid water. The conservative definition of habitability indicates that an Earth-like atmosphere would have the highest probability of habitability.

The zero-albedo assumption incorporated in our model results in a higher equilibrium temperature of the exoplanets. The atmospheric greenhouse effect is responsible for higher surface temperatures than the equilibrium temperature (non-zero albedos) of a planet. The extent of the feedback is strongly dependent on the type and concentrations of greenhouse gases in the atmosphere, along with several other atmospheric feedbacks [70]. Nonetheless, the higher equilibrium temperatures due to assuming zero albedo are a positive shift towards the possible surface temperatures. For example, Earth has an equilibrium temperature of approximately 255 K and a global mean surface temperature of approximately 288 K [71]; by assuming zero albedo, the equilibrium temperature is calculated to be approximately 280 K.

**Figure 8.**
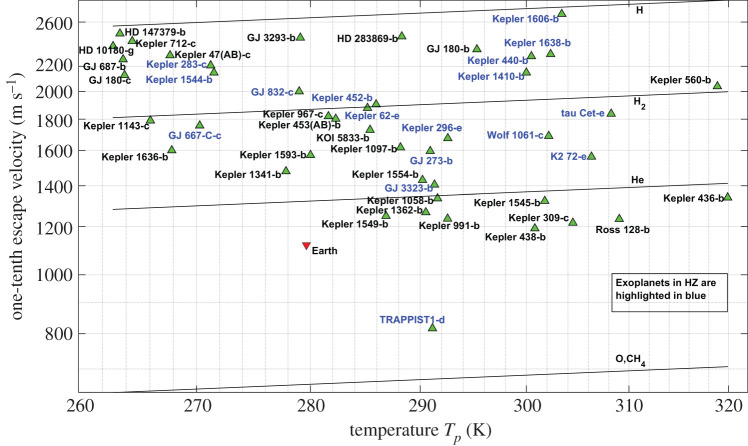
One-tenth of the escape velocity–equilibrium temperature, *T*_*p*_, diagram of the exoplanets of interest for habitability studies. The figure shows a conservative list of exoplanets which could be potentially habitable from the estimations of our model. The names of the exoplanets which are already considered potentially habitable are highlighted in blue. (Online version in colour.)

We find, based on our calculations described above, that 45 known exoplanets satisfy these criteria. They are shown in figure 8 and are listed in electronic supplementary material, table S3. We have excluded in figure 8 the exoplanets that host Jupiter-like atmospheres, with large amounts of hydrogen. These exoplanets could have CH_4_/H_2_O/N_2_/O_2_/CO_2_ as the dominant atmospheric species. This list of potentially habitable exoplanets includes exoplanets with masses ranging from approximately 25 M_e_ (HD 147379 b) to approximately 0.4 M_e_ (TRAPPIST-1 d).

The most recent available listing of 55 potentially habitable exoplanets is presented in the habitable exoplanets catalogue [27] (see electronic supplementary material, table S2). Only 17 of these exoplanets (highlighted in blue in figure 8) have an Earth-like atmosphere. A stable atmosphere is needed for the stability of liquid water, so, from our point of view, only these 17 exoplanets from that list should be potentially habitable.

On the other hand, we have found 28 additional planets, not listed in the habitable exoplanets catalogue [27], that have the potential to host an atmosphere and liquid water, so our list of potentially habitable exoplanets amounts to 45 exoplanets (28 of them different from those listed in [27]). Electronic supplementary material, table S3 contains the 45 exoplanets.

## 4. Discussion

With the advent of new space missions to study and characterize the exoplanets and the increasing rate of discoveries of exoplanets, the significance of finding habitable exoplanets and characterizing their atmospheres is increasing. The discrimination of exoplanets based on the gas species that they can retain in their atmospheres will help to determine the most probable candidates for potential habitability, for further atmospheric composition studies and for photochemical models.

Lack of data on the exoplanets regarding their exosphere, atmospheric structure and composition renders implementing the physically correct Jeans escape a near-impossible task. The lack of atmospheric temperature profiles and compositions can be partially addressed by incorporating transit spectroscopy and radiative transfer models for hot Jupiters and close-in exoplanets. The complex problem of exoplanet exospheres could not be solved for low-mass exoplanets because of their strong dependence on local conditions [15].

We present an atmospheric model capable of estimating the plausible composition of exoplanet atmospheres using the readily available or estimable parameters and with minimal assumptions. Figure 2 shows that the presented model, although primitive, can determine the atmospheric species escaping from atmospheres of Earth-like terrestrial exoplanets. The model we presented is primarily a substitute for Jeans escape until it can be implemented for exoplanets with knowledge of their exospheric parameters and certain physical and chemical conditions of the atmospheres.

Our results suggest that the definition of the HZ around a star should be revisited and that the capacity of the planet to host an Earth-like atmosphere to support the stability of liquid water should be added. Our model is characteristically a straightforward model to estimate plausible atmospheric compositions. In contrast to the existing atmospheric models built on the hydrodynamic escape of hot Jupiter and close-in irradiated exoplanets [15,16,72–74], our model is designed for low-mass, low-irradiated exoplanets. These exoplanets have atmospheric evolution driven by classical thermal escape.

These results could be beneficial for the detection of specific atmospheric species and for biomarker observations of many of the active and planned exoplanet characterization missions such as the Hubble Telescope [75], CHEOPS (CHaracterizing ExOPlanet Satellite) [76], JWST (James Webb Space Telescope) [77], E-ELT (European Extremely Large Telescope) [78], W.M. Keck Observatory [79], Gemini Observatory [80] and CARMENES [81], which are tasked with observing the atmospheres of exoplanets. Many sophisticated exoplanet target lists incorporating complex atmospheric models are available in the literature for the missions mentioned above. Our model results, with the simple kinetic equation, show that it can be used as a preliminary classification method, more specifically for low-mass exoplanets.

The relevance of our results at the present time is emphasized by the increasing number of Earth-sized exoplanets that could follow slow thermal escape. The active and upcoming missions may fall short of characterizing atmospheres of Earth-like exoplanets around Sun-like stars [16]. The transit observations fundamentally favour close-in exoplanets, hot Jupiters and H/He-dominant atmospheres owing to their relatively strong spectral signal strengths [82].

The model can be further improved by including the actual albedo instead of assuming a zero albedo, measured temperature profiles of exoplanets and equilibrium chemistry along with estimations of elemental abundances. Albeit with limitations, the model enables us to estimate the list of atmospheric gases that the exoplanets can retain in their atmospheres and their plausible atmospheres as compared with those observed in the Solar System. Based on the atmospheres observed on the Solar System planets, we list the exoplanets as those with H/He, H_2_O/N_2_/CO_2_ or evaporating/thin silicate atmospheres. We also propose a conservative list of 45 exoplanets with favourable conditions such as temperature and ability to retain the essential life-related gases in their atmospheres for further habitability studies. Further analysis considering the possible escape mechanisms and chemical processes such as photodissociation, sputtering, ion pick-up and suprathermal escape would decisively determine the habitability and atmospheric compositions of these exoplanets.

As exoplanets are discovered continuously, we will keep an updated list of habitable exoplanet targets and possible atmospheric species in a dedicated webpage: https://atmospheres.research.ltu.se/exoplanets_species.php

## Supplementary Material

Supplementary data

## Data accessibility

The datasets analysed in this study are available at the Planetary Habitability Laboratory (PHL), University of Puerto Rico, Arecibo: http://phl.upr.edu/projects/habitable-exoplanets-catalog/data/database and http://exoplanet.eu/. The datasets generated during the current study will evolve with the availability of new data and updates to the exoplanet catalogues. All up-to-date datasets will be made publicly available by the Group of Atmospheric Science, LTU, Sweden: https://atmospheres.research.ltu.se/exoplanets_species.php.

Extended results and tables are available in the electronic supplementary material.
