# Supplementary material for: Atmospheric composition of exoplanets based on the thermal escape of gases and implications for habitability
Source: Proc Math Phys Eng Sci. 2020 Sep 9;476(2241):20200148. doi: 10.1098/rspa.2020.0148 (PMC7544335; doi:10.1098/rspa.2020.0148)
Supplement: Supplementary data [file rspa20200148supp1.docx]

**Atmospheric composition of exoplanets based on the thermal escape of gases and implications on habitability**

Samuel Konatham^1^*, Javier Martin-Torres^1,2,3^, Maria-Paz Zorzano^4,1^

^1^ Group of Atmospheric Science, Department of Computer Science, Electrical and Space Engineering, Luleå University of Technology, Luleå, Sweden

^2^ Instituto Andaluz de Ciencias de la Tierra (CSIC-UGR), Armilla, Granada, Spain

^3^ School of Geosciences, University of Aberdeen, Meston Building, King’s College, Aberdeen, UK

^4^ Centro de Astrobiología (CSIC-INTA), Torrejón de Ardoz, Madrid, Spain

*e-mail: samuel.konatham@ltu.se

**Supplementary data**

**Table S1: Estimations of atmospheric species for exoplanets with reported detections**

|  | Exoplanet | Reported predictions and detections of atmospheric species | Estimations from our model |
| --- | --- | --- | --- |
| 1 | 1RXS 1609 b | [1] CO, H_2_O, K | All gases |
| 2 | 2M 2236+4751 b | [2] CO, H_2_O | All gases |
| 3 | 51 Eri b | [3] CH_4_, H_2_O | All gases |
| 4 | 51 Peg b | [4] CO, H_2_O | Heavier than H |
| 5 | 55 Cnc e | [5] HCN, H_2_O | Heavier than He |
| 6 | GJ 1132 b | [6] CO_2_, H_2_, N_2_, O_2_ | Heavier than He |
| 7 | GJ 1214 b | [7] He | Heavier than H |
| 8 | GJ 3470 b | [8] H | Heavier than H |
| 9 | GJ 436 b | [9,10] CO_2_, CH_4_, H, H_2_O, CO | Heavier than H |
| 10 | GJ 504 b | [11] CH_4_ | All gases |
| 11 | GJ 758 b | [12] CH_4_ | All gases |
| 12 | GU Psc b | [13] CH_4_ | All gases |
| 13 | HAT-P-1 b | [14,15] C, O I, H_2_O, K | Heavier than H |
| 14 | HAT-P-11 b | [16] He | Heavier than H |
| 15 | HAT-P-12 b | [17] Na | Heavier than H |
| 16 | HAT-P-26 | [18] H_2_O | Heavier than He |
| 17 | HD 179949 b | [19] CO, H_2_O | All gases |
| 18 | HD 189733 b | [20] CO_2_, H, H_2_O, Na, C, O I, CO, CH_4_ | All gases |
| 19 | HD 209458 b | [21–23] CO_2_, H, TiO, O_2_, Na, Mg, C, O I, CH_4_, NH_3_, VO, H_2_, HCN, H_2_O, K, CO | Heavier than H |
| 20 | HD 80606 b | [24] K | All gases |
| 21 | HR 8799 b | [25] CO, CH_4_, H_2_O | All gases |
| 22 | HR 8799 c | [26] H_2_O | All gases |
| 23 | ROXs 42B b | [27] CO, H_2_O, K | All gases |
| 24 | Ross 458(AB) c | [28] H_2_, CH_4_, H_2_O, K | All gases |
| 25 | TRAPPIST-1 b | [29] H_2_O | Heavier than He |
| 26 | TRAPPIST-1 c | [29] H_2_O | Heaveir the H |
| 27 | TRAPPIST-1 d | [29] H_2_O | Heavier than He |
| 28 | TRAPPIST-1 e | [29] H_2_O | Heavier than He |
| 29 | TRAPPIST-1 f | [29] H_2_O | Heavier than He |
| 30 | TRAPPIST-1 g | [29] H_2_O | Heavier than H |
| 31 | TRAPPIST-1 h | [29] H_2_O | Heavier than He |
| 32 | WASP-103 b | [30] Na, K | Heavier than H |
| 33 | WASP-107 b | [31] He | Heavier than H |
| 34 | WASP-12 | [15,32] CO_2_, H, TiO, Na, C, O I, He, CO, CH_4_, VO, H_2_, HCN, H_2_O | Heavier than H |
| 35 | WASP-121 b | [33] VO, H_2_O, TiO | Heavier than H |
| 36 | WASP-127 b | [34] Na, H_2_O, Li, K | Heavier than He |
| 37 | WASP-17 b | [35] Na, C, O I, K | Heavier than H |
| 38 | WASP-18 b | [36] H_2_O | All gases |
| 39 | WASP-19 b | [15,37] C, O I, TiO | Heavier than H |
| 40 | WASP-33 b | [38] AlO | All gases |
| 41 | WASP-39 b | [39] H_2_O | Heavier than H |
| 42 | WASP-43 b | [15,40] CO_2_, H_2_O, C, O I, CO, CH_4_, NH_3_ | All gases |
| 43 | WASP-52 b | [41] Na | Heavier than H |
| 44 | WASP-69 b | [42] Na | Heavier than H |
| 45 | WASP-76 b | [43] Na | Heavier than H |
| 46 | WASP-80 b | [44] H, H_2_O, K, Na, He, CO, CH_4_ | All gases |
| 47 | WASP-98 b | [45] VO, TiO | All gases |
| 48 | WISE 0458+6434 b | [46] CH_4_, H_2_O | All gases |
| 49 | WISE 1217+16 A b | [47] H_2_, CH_4_, H_2_O | All gases |
| 50 | XO-1 b | [15] C, O I | All gases |
| 51 | XO-2N b | [48] Na, K | All gases |
| 52 | beta Pic b | [49] H | All gases |
| 53 | kappa And b | [50] H_2_O | All gases |
| 54 | tau Boo b | [51] H_2_O | All gases |

**Table S2: List of Potentially habitable exoplanets listed in The Habitable Exoplanets catalogue.**[52]

|  | **Exoplanets** |
| --- | --- |
| 1 | [Teegarden's Star b](http://exoplanetarchive.ipac.caltech.edu/cgi-bin/DisplayOverview/nph-DisplayOverview?objname=Teegarden's%20Star%20b&type=CONFIRMED_PLANET) |
| 2 | [K2-72 e](http://exoplanetarchive.ipac.caltech.edu/cgi-bin/DisplayOverview/nph-DisplayOverview?objname=K2-72%20e&type=CONFIRMED_PLANET) |
| 3 | [GJ 3323 b](http://exoplanetarchive.ipac.caltech.edu/cgi-bin/DisplayOverview/nph-DisplayOverview?objname=GJ%203323%20b&type=CONFIRMED_PLANET) |
| 4 | [TRAPPIST-1 d](http://exoplanetarchive.ipac.caltech.edu/cgi-bin/DisplayOverview/nph-DisplayOverview?objname=TRAPPIST-1%20d&type=CONFIRMED_PLANET) |
| 5 | [GJ 1061 c](http://exoplanetarchive.ipac.caltech.edu/cgi-bin/DisplayOverview/nph-DisplayOverview?objname=GJ%201061%20c&type=CONFIRMED_PLANET) |
| 6 | [TRAPPIST-1 e](http://exoplanetarchive.ipac.caltech.edu/cgi-bin/DisplayOverview/nph-DisplayOverview?objname=TRAPPIST-1%20e&type=CONFIRMED_PLANET) |
| 7 | [GJ 667 C f](http://exoplanetarchive.ipac.caltech.edu/cgi-bin/DisplayOverview/nph-DisplayOverview?objname=GJ%20667%20C%20f&type=CONFIRMED_PLANET) |
| 8 | [Proxima Cen b](http://exoplanetarchive.ipac.caltech.edu/cgi-bin/DisplayOverview/nph-DisplayOverview?objname=Proxima%20Cen%20b&type=CONFIRMED_PLANET) |
| 9 | [Kepler-442 b](http://exoplanetarchive.ipac.caltech.edu/cgi-bin/DisplayOverview/nph-DisplayOverview?objname=Kepler-442%20b&type=CONFIRMED_PLANET) |
| 10 | [GJ 273 b](http://exoplanetarchive.ipac.caltech.edu/cgi-bin/DisplayOverview/nph-DisplayOverview?objname=GJ%20273%20b&type=CONFIRMED_PLANET) |
| 11 | [GJ 1061 d](http://exoplanetarchive.ipac.caltech.edu/cgi-bin/DisplayOverview/nph-DisplayOverview?objname=GJ%201061%20d&type=CONFIRMED_PLANET) |
| 12 | [Wolf 1061 c](http://exoplanetarchive.ipac.caltech.edu/cgi-bin/DisplayOverview/nph-DisplayOverview?objname=Wolf%201061%20c&type=CONFIRMED_PLANET) |
| 13 | [GJ 667 C c](http://exoplanetarchive.ipac.caltech.edu/cgi-bin/DisplayOverview/nph-DisplayOverview?objname=GJ%20667%20C%20c&type=CONFIRMED_PLANET) |
| 14 | [tau Cet e](http://exoplanetarchive.ipac.caltech.edu/cgi-bin/DisplayOverview/nph-DisplayOverview?objname=tau%20Cet%20e&type=CONFIRMED_PLANET) |
| 15 | [Kepler-1229 b](http://exoplanetarchive.ipac.caltech.edu/cgi-bin/DisplayOverview/nph-DisplayOverview?objname=Kepler-1229%20b&type=CONFIRMED_PLANET) |
| 16 | [GJ 667 C e](http://exoplanetarchive.ipac.caltech.edu/cgi-bin/DisplayOverview/nph-DisplayOverview?objname=GJ%20667%20C%20e&type=CONFIRMED_PLANET) |
| 17 | [TRAPPIST-1 f](http://exoplanetarchive.ipac.caltech.edu/cgi-bin/DisplayOverview/nph-DisplayOverview?objname=TRAPPIST-1%20f&type=CONFIRMED_PLANET) |
| 18 | [Teegarden's Star c](http://exoplanetarchive.ipac.caltech.edu/cgi-bin/DisplayOverview/nph-DisplayOverview?objname=Teegarden's%20Star%20c&type=CONFIRMED_PLANET) |
| 19 | [Kepler-62 f](http://exoplanetarchive.ipac.caltech.edu/cgi-bin/DisplayOverview/nph-DisplayOverview?objname=Kepler-62%20f&type=CONFIRMED_PLANET) |
| 20 | [TRAPPIST-1 g](http://exoplanetarchive.ipac.caltech.edu/cgi-bin/DisplayOverview/nph-DisplayOverview?objname=TRAPPIST-1%20g&type=CONFIRMED_PLANET) |
| 21 | [Kepler-186 f](http://exoplanetarchive.ipac.caltech.edu/cgi-bin/DisplayOverview/nph-DisplayOverview?objname=Kepler-186%20f&type=CONFIRMED_PLANET) |
| 22 | [Kepler-452 b](http://exoplanetarchive.ipac.caltech.edu/cgi-bin/DisplayOverview/nph-DisplayOverview?objname=Kepler-452%20b&type=CONFIRMED_PLANET) |
| 23 | [Kepler-62 e](http://exoplanetarchive.ipac.caltech.edu/cgi-bin/DisplayOverview/nph-DisplayOverview?objname=Kepler-62%20e&type=CONFIRMED_PLANET) |
| 24 | [Kepler-1652 b](http://exoplanetarchive.ipac.caltech.edu/cgi-bin/DisplayOverview/nph-DisplayOverview?objname=Kepler-1652%20b&type=CONFIRMED_PLANET) |
| 25 | [Kepler-1544 b](http://exoplanetarchive.ipac.caltech.edu/cgi-bin/DisplayOverview/nph-DisplayOverview?objname=Kepler-1544%20b&type=CONFIRMED_PLANET) |
| 26 | [K2-3 d](http://exoplanetarchive.ipac.caltech.edu/cgi-bin/DisplayOverview/nph-DisplayOverview?objname=K2-3%20d&type=CONFIRMED_PLANET) |
| 27 | [Kepler-296 e](http://exoplanetarchive.ipac.caltech.edu/cgi-bin/DisplayOverview/nph-DisplayOverview?objname=Kepler-296%20e&type=CONFIRMED_PLANET) |
| 28 | [Kepler-283 c](http://exoplanetarchive.ipac.caltech.edu/cgi-bin/DisplayOverview/nph-DisplayOverview?objname=Kepler-283%20c&type=CONFIRMED_PLANET) |
| 29 | [Kepler-1410 b](http://exoplanetarchive.ipac.caltech.edu/cgi-bin/DisplayOverview/nph-DisplayOverview?objname=Kepler-1410%20b&type=CONFIRMED_PLANET) |
| 30 | [Kepler-1638 b](http://exoplanetarchive.ipac.caltech.edu/cgi-bin/DisplayOverview/nph-DisplayOverview?objname=Kepler-1638%20b&type=CONFIRMED_PLANET) |
| 31 | [K2-296 b](http://exoplanetarchive.ipac.caltech.edu/cgi-bin/DisplayOverview/nph-DisplayOverview?objname=K2-296%20b&type=CONFIRMED_PLANET) |
| 32 | [Kepler-296 f](http://exoplanetarchive.ipac.caltech.edu/cgi-bin/DisplayOverview/nph-DisplayOverview?objname=Kepler-296%20f&type=CONFIRMED_PLANET) |
| 33 | [Kepler-705 b](http://exoplanetarchive.ipac.caltech.edu/cgi-bin/DisplayOverview/nph-DisplayOverview?objname=Kepler-705%20b&type=CONFIRMED_PLANET) |
| 34 | [Kepler-440 b](http://exoplanetarchive.ipac.caltech.edu/cgi-bin/DisplayOverview/nph-DisplayOverview?objname=Kepler-440%20b&type=CONFIRMED_PLANET) |
| 35 | [Kepler-1653 b](http://exoplanetarchive.ipac.caltech.edu/cgi-bin/DisplayOverview/nph-DisplayOverview?objname=Kepler-1653%20b&type=CONFIRMED_PLANET) |
| 36 | [GJ 832 c](http://exoplanetarchive.ipac.caltech.edu/cgi-bin/DisplayOverview/nph-DisplayOverview?objname=GJ%20832%20c&type=CONFIRMED_PLANET) |
| 37 | [Kepler-1606 b](http://exoplanetarchive.ipac.caltech.edu/cgi-bin/DisplayOverview/nph-DisplayOverview?objname=Kepler-1606%20b&type=CONFIRMED_PLANET) |
| 38 | [Kepler-1090 b](http://exoplanetarchive.ipac.caltech.edu/cgi-bin/DisplayOverview/nph-DisplayOverview?objname=Kepler-1090%20b&type=CONFIRMED_PLANET) |
| 39 | [Kepler-61 b](http://exoplanetarchive.ipac.caltech.edu/cgi-bin/DisplayOverview/nph-DisplayOverview?objname=Kepler-61%20b&type=CONFIRMED_PLANET) |
| 40 | [Kepler-443 b](http://exoplanetarchive.ipac.caltech.edu/cgi-bin/DisplayOverview/nph-DisplayOverview?objname=Kepler-443%20b&type=CONFIRMED_PLANET) |
| 41 | [K2-18 b](http://exoplanetarchive.ipac.caltech.edu/cgi-bin/DisplayOverview/nph-DisplayOverview?objname=K2-18%20b&type=CONFIRMED_PLANET) |
| 42 | [Kepler-22 b](http://exoplanetarchive.ipac.caltech.edu/cgi-bin/DisplayOverview/nph-DisplayOverview?objname=Kepler-22%20b&type=CONFIRMED_PLANET) |
| 43 | [K2-9 b](http://exoplanetarchive.ipac.caltech.edu/cgi-bin/DisplayOverview/nph-DisplayOverview?objname=K2-9%20b&type=CONFIRMED_PLANET) |
| 44 | [Kepler-26 e](http://exoplanetarchive.ipac.caltech.edu/cgi-bin/DisplayOverview/nph-DisplayOverview?objname=Kepler-26%20e&type=CONFIRMED_PLANET) |
| 45 | [Kepler-1552 b](http://exoplanetarchive.ipac.caltech.edu/cgi-bin/DisplayOverview/nph-DisplayOverview?objname=Kepler-1552%20b&type=CONFIRMED_PLANET) |
| 46 | [Kepler-1540 b](http://exoplanetarchive.ipac.caltech.edu/cgi-bin/DisplayOverview/nph-DisplayOverview?objname=Kepler-1540%20b&type=CONFIRMED_PLANET) |
| 47 | [LHS 1140 b](http://exoplanetarchive.ipac.caltech.edu/cgi-bin/DisplayOverview/nph-DisplayOverview?objname=LHS%201140%20b&type=CONFIRMED_PLANET) |
| 48 | [Kepler-1632 b](http://exoplanetarchive.ipac.caltech.edu/cgi-bin/DisplayOverview/nph-DisplayOverview?objname=Kepler-1632%20b&type=CONFIRMED_PLANET) |
| 49 | [HD 40307 g](http://exoplanetarchive.ipac.caltech.edu/cgi-bin/DisplayOverview/nph-DisplayOverview?objname=HD%2040307%20g&type=CONFIRMED_PLANET) |
| 50 | [GJ 163 c](http://exoplanetarchive.ipac.caltech.edu/cgi-bin/DisplayOverview/nph-DisplayOverview?objname=GJ%20163%20c&type=CONFIRMED_PLANET) |
| 51 | [Kepler-298 d](http://exoplanetarchive.ipac.caltech.edu/cgi-bin/DisplayOverview/nph-DisplayOverview?objname=Kepler-298%20d&type=CONFIRMED_PLANET) |
| 52 | [K2-288 B b](http://exoplanetarchive.ipac.caltech.edu/cgi-bin/DisplayOverview/nph-DisplayOverview?objname=K2-288%20B%20b&type=CONFIRMED_PLANET) |
| 53 | [GJ 3293 d](http://exoplanetarchive.ipac.caltech.edu/cgi-bin/DisplayOverview/nph-DisplayOverview?objname=GJ%203293%20d&type=CONFIRMED_PLANET) |
| 54 | [Kepler-174 d](http://exoplanetarchive.ipac.caltech.edu/cgi-bin/DisplayOverview/nph-DisplayOverview?objname=Kepler-174%20d&type=CONFIRMED_PLANET) |
| 55 | [GJ 357 d](http://exoplanetarchive.ipac.caltech.edu/cgi-bin/DisplayOverview/nph-DisplayOverview?objname=GJ%20357%20d&type=CONFIRMED_PLANET) |

**Table S3: Potentially habitable exoplanets derived from our model**

|  | **Exoplanet** | **Me** |
| --- | --- | --- |
| 1 | GJ-180-b | 8.3 |
| 2 | GJ-180-c | 6.4 |
| 3 | GJ-273-b | 2.89 |
| 4 | GJ-667-C-c | 3.8 |
| 5 | GJ-687-b | 18.44 |
| 6 | GJ-832-c | 5.4 |
| 7 | GJ-3293-b | 23.55 |
| 8 | GJ-3323-b | 2.02 |
| 9 | HD-10180-g | 21.41 |
| 10 | HD-147379-b | 24.7 |
| 11 | HD-283869-b | 9.49 |
| 12 | K2-72-e | 2.73 |
| 13 | Kepler-47(AB)-c | 19.26 |
| 14 | Kepler-62-e | 4.54 |
| 15 | Kepler-283-c | 7.04 |
| 16 | Kepler-296-e | 3.32 |
| 17 | Kepler-309-c | 2.97 |
| 18 | Kepler-436-b | 3.91 |
| 19 | Kepler-438-b | 1.27 |
| 20 | Kepler-440-b | 7.75 |
| 21 | Kepler-452-b | 4.72 |
| 22 | Kepler-453(AB)-b | 16 |
| 23 | Kepler-560-b | 5.68 |
| 24 | Kepler-712-c | 22.67 |
| 25 | Kepler-967-c | 9.66 |
| 26 | Kepler-991-b | 3.1 |
| 27 | Kepler-1058-b | 3.87 |
| 28 | Kepler-1097-b | 6.82 |
| 29 | Kepler-1143-c | 9.22 |
| 30 | Kepler-1341-b | 5.22 |
| 31 | Kepler-1362-b | 3.33 |
| 32 | Kepler-1410-b | 6.56 |
| 33 | Kepler-1544-b | 6.56 |
| 34 | Kepler-1545-b | 3.77 |
| 35 | Kepler-1549-b | 3.19 |
| 36 | Kepler-1554-b | 4.74 |
| 37 | Kepler-1593-b | 6.26 |
| 38 | Kepler-1606-b | 11.88 |
| 39 | Kepler-1636-b | 6.61 |
| 40 | Kepler-1638-b | 7.92 |
| 41 | KOI-5833-b | 8.29 |
| 42 | Ross-128-b | 1.4 |
| 43 | tau-Cet-e | 4.29 |
| 44 | TRAPPIST-1-d | 0.41 |
| 45 | Wolf-1061-c | 3.4 |

**References**

1. Lafrenière D, Jayawardhana R, van Kerkwijk MH. 2008 Direct Imaging and Spectroscopy of a Planetary-Mass Candidate Companion to a Young Solar Analog. *Astrophys. J.* **689**, L153–L156. (doi:10.1086/595870)

2. Bowler BP *et al.* 2016 PLANETS AROUND LOW-MASS STARS (PALMS). VI. DISCOVERY OF A REMARKABLY RED PLANETARY-MASS COMPANION TO THE AB DOR MOVING GROUP CANDIDATE 2MASS J22362452+4751425. *Astron. J.* **153**, 18. (doi:10.3847/1538-3881/153/1/18)

3. Macintosh B *et al.* 2015 Discovery and spectroscopy of the young jovian planet 51 Eri b with the Gemini Planet Imager. *Science (80-. ).* **350**, 64–67. (doi:10.1126/science.aac5891)

4. Birkby JL, Kok RJ De, Brogi M, Schwarz H, Snellen IAG. 2017 Discovery of Water at High Spectral Resolution in the Atmosphere of 51 Peg b. *Astron. J.* **153**, 138. (doi:10.3847/1538-3881/aa5c87)

5. Esteves LJ, de Mooij EJW, Jayawardhana R, Watson C, de Kok R. 2017 A Search for Water in a Super-Earth Atmosphere: High-resolution Optical Spectroscopy of 55Cancri e. *Astron. J.* **153**, 268. (doi:10.3847/1538-3881/aa7133)

6. Schaefer L, Wordsworth RD, Berta-Thompson Z, Sasselov D. 2016 PREDICTIONS OF THE ATMOSPHERIC COMPOSITION OF GJ 1132b. *Astrophys. J.* **829**, 63. (doi:10.3847/0004-637X/829/2/63)

7. Crossfield IJM, Barman T, Hansen B, Frewen S. 2019 An Upper Limit on He Absorption in GJ 1214b. *Res. Notes AAS* **3**, 24. (doi:10.3847/2515-5172/ab01b8)

8. Bourrier V *et al.* 2018 Hubble PanCET: an extended upper atmosphere of neutral hydrogen around the warm Neptune GJ 3470b. *Astron. Astrophys.* **620**, A147. (doi:10.1051/0004-6361/201833675)

9. Madhusudhan N, Seager S. 2011 HIGH METALLICITY AND NON-EQUILIBRIUM CHEMISTRY IN THE DAYSIDE ATMOSPHERE OF HOT-NEPTUNE GJ 436b. *Astrophys. J.* **729**, 41. (doi:10.1088/0004-637X/729/1/41)

10. Kulow JR, France K, Linsky J, Parke Loyd RO. 2014 LYα TRANSIT SPECTROSCOPY AND THE NEUTRAL HYDROGEN TAIL OF THE HOT NEPTUNE GJ 436b. *Astrophys. J.* **786**, 132. (doi:10.1088/0004-637X/786/2/132)

11. Janson M *et al.* 2013 DIRECT IMAGING DETECTION OF METHANE IN THE ATMOSPHERE OF GJ 504 b. *Astrophys. J.* **778**, L4. (doi:10.1088/2041-8205/778/1/L4)

12. Janson M *et al.* 2011 NEAR-INFRARED MULTI-BAND PHOTOMETRY OF THE SUBSTELLAR COMPANION GJ 758 B ∗. **85**, 1–6. (doi:10.1088/0004-637X/728/2/85)

13. Naud M-E *et al.* 2014 DISCOVERY OF A WIDE PLANETARY-MASS COMPANION TO THE YOUNG M3 STAR GU PSC. *Astrophys. J.* **787**, 5. (doi:10.1088/0004-637X/787/1/5)

14. Wakeford HR *et al.* 2013 HST hot Jupiter transmission spectral survey: detection of water in HAT-P-1b from WFC3 near-IR spatial scan observations. *Mon. Not. R. Astron. Soc.* **435**, 3481–3493. (doi:10.1093/mnras/stt1536)

15. Benneke B. 2015 Strict Upper Limits on the Carbon-to-Oxygen Ratios of Eight Hot Jupiters from Self-Consistent Atmospheric Retrieval. , 1–19.

16. Mansfield M *et al.* 2018 Detection of Helium in the Atmosphere of the Exo-Neptune HAT-P-11b. *Astrophys. J.* **868**, L34. (doi:10.3847/2041-8213/aaf166)

17. Deibert EK, de Mooij EJW, Jayawardhana R, Fortney JJ, Brogi M, Rustamkulov Z, Tamura M. 2019 High-resolution Transit Spectroscopy of Warm Saturns. *Astron. J.* **157**, 58. (doi:10.3847/1538-3881/aaf56b)

18. Wakeford HR *et al.* 2017 HAT-P-26b: A Neptune-mass exoplanet with a well-constrained heavy element abundance. *Science (80-. ).* **356**, 628–631. (doi:10.1126/science.aah4668)

19. Brogi M, de Kok RJ, Birkby JL, Schwarz H, Snellen IAG. 2014 Carbon monoxide and water vapor in the atmosphere of the non-transiting exoplanet HD 179949 b. *Astron. Astrophys.* **565**, A124. (doi:10.1051/0004-6361/201423537)

20. Allart R, Lovis C, Pino L, Wyttenbach A, Ehrenreich D, Pepe F. 2017 Search for water vapor in the high-resolution transmission spectrum of HD 189733b in the visible. *Astron. Astrophys.* **606**, A144. (doi:10.1051/0004-6361/201730814)

21. Vidal-Madjar A *et al.* 2004 Detection of Oxygen and Carbon in the Hydrodynamically Escaping Atmosphere of the Extrasolar Planet HD 209458b. *Astrophys. J.* **604**, L69–L72. (doi:10.1086/383347)

22. Vidal-Madjar A *et al.* 2013 Magnesium in the atmosphere of the planet HD 209458 b: observations of the thermosphere-exosphere transition region. *Astron. Astrophys.* **560**, A54. (doi:10.1051/0004-6361/201322234)

23. MacDonald RJ, Madhusudhan N. 2017 HD 209458b in new light: evidence of nitrogen chemistry, patchy clouds and sub-solar water. *Mon. Not. R. Astron. Soc.* **469**, 1979–1996. (doi:10.1093/mnras/stx804)

24. Colón KD, Ford EB, Redfield S, Fortney JJ, Shabram M, Deeg HJ, Mahadevan S. 2012 Probing potassium in the atmosphere of HD 80606b with tunable filter transit spectrophotometry from the Gran Telescopio Canarias. *Mon. Not. R. Astron. Soc.* **419**, 2233–2250. (doi:10.1111/j.1365-2966.2011.19878.x)

25. Barman TS, Konopacky QM, Macintosh B, Marois C. 2015 SIMULTANEOUS DETECTION OF WATER, METHANE, AND CARBON MONOXIDE IN THE ATMOSPHERE OF EXOPLANET HR 8799 b. *Astrophys. J.* **804**, 61. (doi:10.1088/0004-637X/804/1/61)

26. Wang J, Mawet D, Fortney JJ, Hood C, Morley C V, Benneke B. 2018 Detecting Water in the Atmosphere of HR 8799 c with L -band High-dispersion Spectroscopy Aided by Adaptive Optics. *Astron. J.* **156**, 272. (doi:10.3847/1538-3881/aae47b)

27. Daemgen S *et al.* 2017 Mid-infrared characterization of the planetary-mass companion ROXs 42B b. *Astron. Astrophys.* **601**, A65. (doi:10.1051/0004-6361/201629949)

28. Burgasser AJ *et al.* 2010 CLOUDS IN THE COLDEST BROWN DWARFS: FIRE SPECTROSCOPY OF ROSS 458C. *Astrophys. J.* **725**, 1405–1420. (doi:10.1088/0004-637X/725/2/1405)

29. Bourrier V *et al.* 2017 Temporal Evolution of the High-energy Irradiation and Water Content of TRAPPIST-1 Exoplanets. *Astron. J.* **154**, 121. (doi:10.3847/1538-3881/aa859c)

30. Lendl M, Cubillos PE, Hagelberg J, Müller A, Juvan I, Fossati L. 2017 Signs of strong Na and K absorption in the transmission spectrum of WASP-103b. *Astron. Astrophys.* **606**, A18. (doi:10.1051/0004-6361/201731242)

31. Allart R *et al.* 2019 High-resolution confirmation of an extended helium atmosphere around WASP-107b. *Astron. Astrophys.* **623**, A58. (doi:10.1051/0004-6361/201834917)

32. Jensen AG, Cauley PW, Redfield S, Cochran WD, Endl M. 2018 Hydrogen and Sodium Absorption in the Optical Transmission Spectrum of WASP-12b. *Astron. J.* **156**, 154. (doi:10.3847/1538-3881/aadca7)

33. Evans TM *et al.* 2018 An Optical Transmission Spectrum for the Ultra-hot Jupiter WASP-121b Measured with the Hubble Space Telescope. *Astron. J.* **156**, 283. (doi:10.3847/1538-3881/aaebff)

34. Chen G *et al.* 2018 The GTC exoplanet transit spectroscopy survey IX. Detection of haze, Na, K, and Li in the super-Neptune WASP-127b. *Astron. Astrophys.* **616**, A145. (doi:10.1051/0004-6361/201833033)

35. Sedaghati E *et al.* 2016 Potassium detection in the clear atmosphere of a hot-Jupiter: FORS2 transmission spectroscopy of WASP-17b. *Astron. Astrophys.* **596**, A47. (doi:10.1051/0004-6361/201629090)

36. Arcangeli J *et al.* 2018 H − Opacity and Water Dissociation in the Dayside Atmosphere of the Very Hot Gas Giant WASP-18b. *Astrophys. J.* **855**, L30. (doi:10.3847/2041-8213/aab272)

37. Sedaghati E, Boffin HMJ, MacDonald RJ, Gandhi S, Madhusudhan N, Gibson NP, Oshagh M, Claret A, Rauer H. 2017 Detection of titanium oxide in the atmosphere of a hot Jupiter. *Nature* **549**, 238–241. (doi:10.1038/nature23651)

38. von Essen C, Mallonn M, Welbanks L, Madhusudhan N, Pinhas A, Bouy H, Weis Hansen P. 2019 An optical transmission spectrum of the ultra-hot Jupiter WASP-33 b. *Astron. Astrophys.* **622**, A71. (doi:10.1051/0004-6361/201833837)

39. Wakeford HR *et al.* 2017 The Complete Transmission Spectrum of WASP-39b with a Precise Water Constraint. *Astron. J.* **155**, 29. (doi:10.3847/1538-3881/aa9e4e)

40. Feng YK, Line MR, Fortney JJ, Stevenson KB, Bean J, Kreidberg L, Parmentier V. 2016 THE IMPACT OF NON-UNIFORM THERMAL STRUCTURE ON THE INTERPRETATION OF EXOPLANET EMISSION SPECTRA. *Astrophys. J.* **829**, 52. (doi:10.3847/0004-637X/829/1/52)

41. Alam MK *et al.* 2018 The HST PanCET Program: Hints of Na i and Evidence of a Cloudy Atmosphere for the Inflated Hot Jupiter WASP-52b. *Astron. J.* **156**, 298. (doi:10.3847/1538-3881/aaee89)

42. Nortmann L *et al.* 2018 Ground-based detection of an extended helium atmosphere in the Saturn-mass exoplanet WASP-69b. *Science (80-. ).* **362**, 1388–1391. (doi:10.1126/science.aat5348)

43. Seidel J V. *et al.* 2019 Hot Exoplanet Atmospheres Resolved with Transit Spectroscopy (HEARTS): II. A broadened sodium feature on the ultra-hot giant WASP-76b. *Astron. Astrophys.* **623**, 1–11. (doi:10.1051/0004-6361/201834776)

44. Sedaghati E, Boffin HMJ, Delrez L, Gillon M, Csizmadia S, Smith AMS, Rauer H. 2017 Probing the atmosphere of a sub-Jovian planet orbiting a cool dwarf. *Mon. Not. R. Astron. Soc.* **468**, 3123–3134. (doi:10.1093/mnras/stx646)

45. Mancini L, Giordano M, Mollière P, Southworth J, Brahm R, Ciceri S, Henning T. 2016 An optical transmission spectrum of the transiting hot Jupiter in the metal-poor WASP-98 planetary system. *Mon. Not. R. Astron. Soc.* **461**, 1053–1061. (doi:10.1093/mnras/stw1386)

46. Mainzer A *et al.* 2011 THE FIRST ULTRA-COOL BROWN DWARF DISCOVERED BY THE WIDE-FIELD INFRARED SURVEY EXPLORER. *Astrophys. J.* **726**, 30. (doi:10.1088/0004-637X/726/1/30)

47. Leggett SK, Liu MC, Dupuy TJ, Morley C V, Marley MS, Saumon D. 2013 RESOLVED SPECTROSCOPY OF THE T8.5 AND Y0-0.5 BINARY WISEPC J121756.91+162640.2AB. *Astrophys. J.* **780**, 62. (doi:10.1088/0004-637X/780/1/62)

48. Sing DK *et al.* 2012 GTC OSIRIS transiting exoplanet atmospheric survey: Detection of sodium in XO-2b from differential long-slit spectroscopy. *Mon. Not. R. Astron. Soc.* **426**, 1663–1670. (doi:10.1111/j.1365-2966.2012.21938.x)

49. Wilson PA, Kerr R, Lecavelier des Etangs A, Bourrier V, Vidal-Madjar A, Kiefer F, Snellen IAG. 2019 Detection of nitrogen gas in the β Pictoris circumstellar disc. *Astron. Astrophys.* **621**, A121. (doi:10.1051/0004-6361/201834346)

50. Todorov KO, Line MR, Pineda JE, Meyer MR, Quanz SP, Hinkley S, Fortney JJ. 2016 THE WATER ABUNDANCE OF THE DIRECTLY IMAGED SUBSTELLAR COMPANION κ AND b RETRIEVED FROM A NEAR INFRARED SPECTRUM. *Astrophys. J.* **823**, 14. (doi:10.3847/0004-637x/823/1/14)

51. Lockwood AC, Johnson JA, Bender CF, Carr JS, Barman T, Richert AJW, Blake GA. 2014 NEAR-IR direct detection of water vapor in tau Boötis b. *Astrophys. J. Lett.* **783**. (doi:10.1088/2041-8205/783/2/L29)

52. PHL@UPRA. In press. The Habitable Exoplanets Catalog - Planetary Habitability Laboratory @ UPR Arecibo. *Habitable Exopl. Cat.* See http://phl.upr.edu/projects/habitable-exoplanets-catalog.
